# Severe Hyperkeratosis Associated With Chronic Lower-Extremity Lymphedema Complicated by Cellulitis: A Case Report

**DOI:** 10.7759/cureus.113965

**Published:** 2026-08-04

**Authors:** Ashmeet Bedi, John Sawires, Alise Leiboff, Akul Ramayani, Rajan Dey

**Affiliations:** 1 Medicine, Lake Erie College of Osteopathic Medicine, Erie, USA; 2 Anesthesiology and Critical Care, Lake Erie College of Osteopathic Medicine, Erie, USA; 3 Geriatrics, Andrus on Hudson, Hastings-on-Hudson, USA

**Keywords:** case report, cellulitis, hyperkeratosis, lymphedema, polymicrobial, vascular

## Abstract

Elephantiasis nostras verrucosa (ENV) is a rare, end-stage complication of chronic nonfilarial lymphedema, marked by progressive dermal fibrosis, hyperkeratosis, and verrucous skin changes, which increase susceptibility to recurrent infections and chronic wounds. We present a 77-year-old man with longstanding bilateral lower-extremity lymphedema, severe hyperkeratosis, malodorous drainage, and polymicrobial wound infection. Examination revealed extensive verrucous lesions with worsening edema and drainage from chronic venous stasis wounds, along with hyperkeratotic changes consistent with ENV. Management included intravenous cefepime and a multidisciplinary approach using the TIME framework, with involvement of infectious disease, wound care, and vascular surgery. He was discharged with a structured wound care plan, ongoing antimicrobials, and coordinated outpatient follow-up. This case underscores the dermatologic and infectious complications of advanced lymphedema and the microbiologic complexity of chronic wounds. Effective management requires early recognition, culture-guided therapy, edema control, structured wound management, and optimization of comorbidities to improve healing, reduce recurrence, and minimize healthcare burden.

## Introduction

Chronic lymphedema is progressive and caused by impaired lymphatic drainage. It can be classified as primary (congenital or developmental lymphatic anomalies) or secondary (acquired trauma, surgery, malignancy, or systemic disease) [1]. Over time, this fluid stasis triggers chronic inflammation, leading to adipose tissue deposition and fibrosis. Without targeted therapy, patients are at increased risk for recurrent infections and significant morbidity [2]. Longstanding lymphatic stasis in chronic lymphedema can lead to advanced cutaneous changes termed elephantiasis nostras verrucosa (ENV). The dermatologic manifestations of this condition are characterized by epidermal hyperkeratosis, papillomatosis, verrucous nodules, and “woody” dermal fibrosis of the affected area [3]. Changes include nonpitting edema, cobblestoned papules, and thickened, lichenified skin that underlie verrucous overgrowths [4].

Bacterial and fungal colonization can be worse in lymphedema because impaired lymphatic drainage, stagnant protein-rich fluid, and disrupted skin barriers increase susceptibility to infection. Cellulitis is common in patients with lymphedema, with large cohort studies reporting that approximately 30-40% experience at least one episode and 20-25% have recurrent cellulitis [5]. Chronic venous and lymphatic wounds are typically colonized or infected by a polymicrobial community, most commonly *Staphylococcus aureus*, beta-hemolytic streptococci, *Pseudomonas aeruginosa*, and *Proteus* species [6]. Systemic risk factors, like obesity, specifically markedly worsen lymphatic function and promote fibrosis. LIMPRINT data show that class III obesity (BMI >40) nearly triples the risk of advanced lymphedema and recurrent cellulitis [7].

Here, we present a patient with advanced bilateral lower-extremity lymphedema complicated by ENV and chronic wounds, highlighting diagnostic considerations, culture interpretation, and a pragmatic multidisciplinary management strategy. This case illustrates (1) the dermatologic end-stage morphology of longstanding lymphedema, (2) the risks and microbiology of secondary infection, and (3) the need for coordinated wound care, infectious disease, and vascular/primary care follow-up to reduce readmissions and cost. This report complies with the Health Insurance Portability and Accountability Act; patient authorization for publication was obtained, and informed consent was obtained and documented. Institutional Review Board approval was not required for this case report involving three or fewer patients.

## Case presentation

Patient information

**Figure 1:**
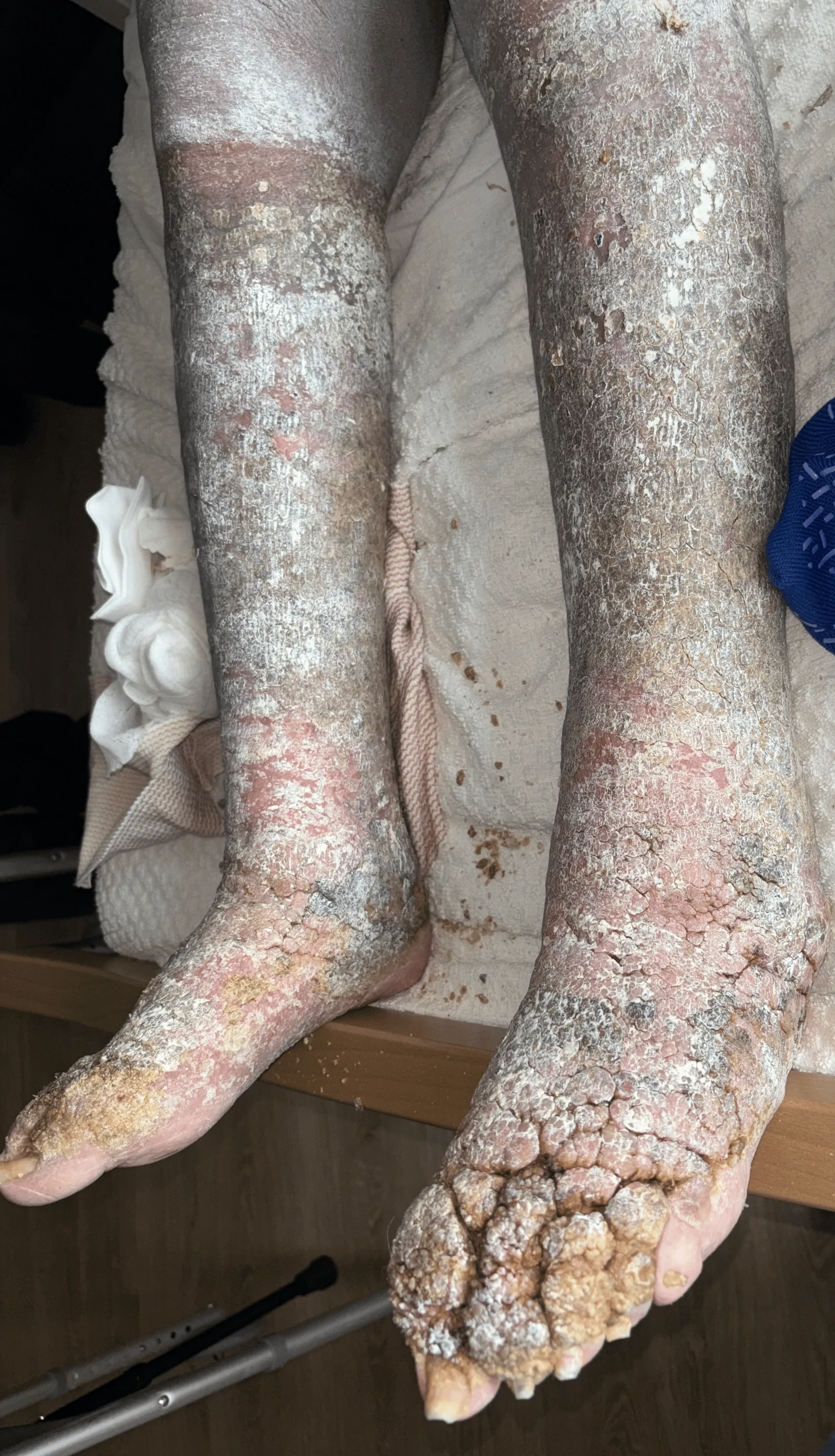
Diffuse hyperkeratosis, scaling, and chronic skin changes in the setting of longstanding lymphedema

**Figure 2:**
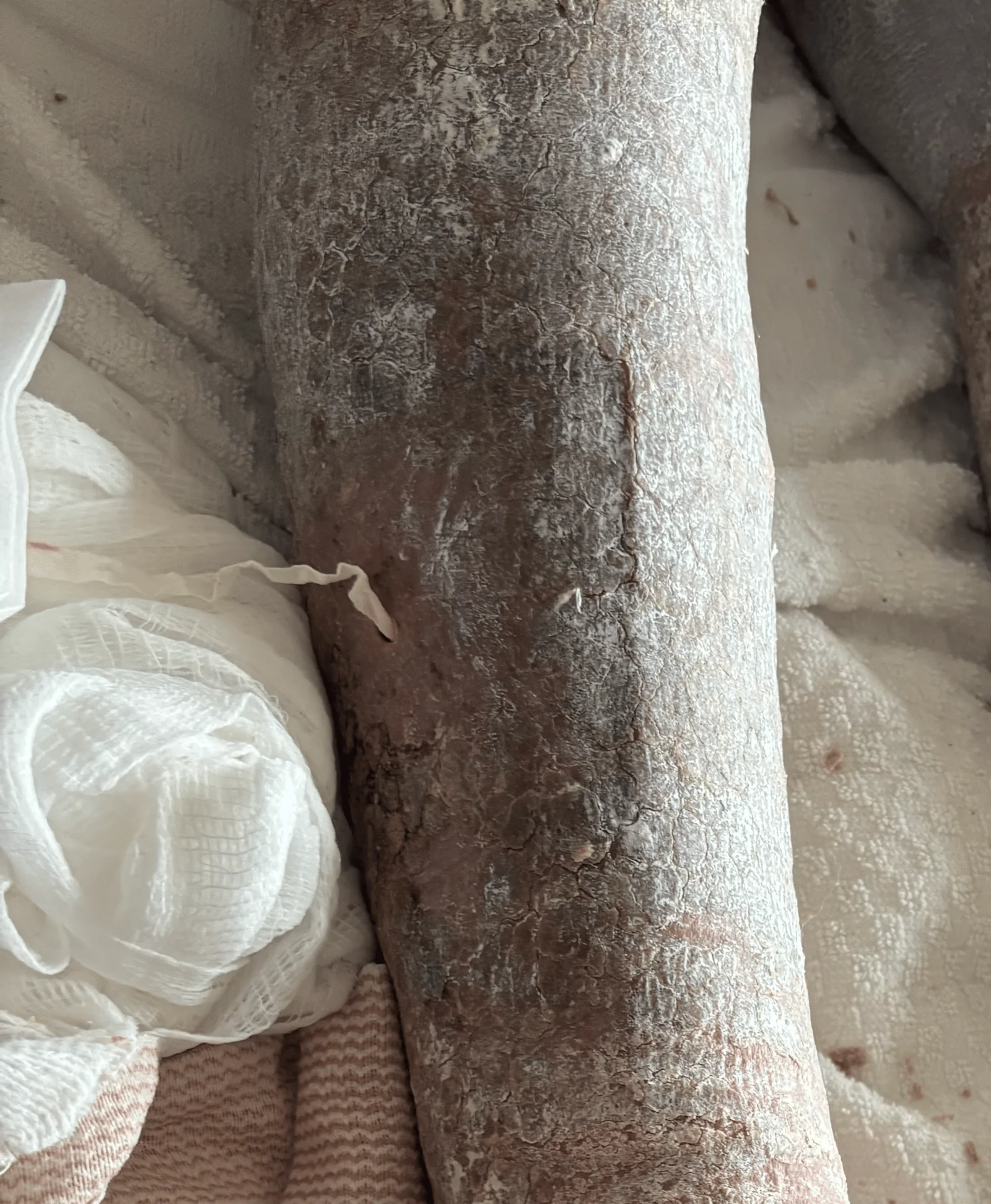
Open ulceration of the right lower leg with sterile ribbon gauze packing within the wound cavity, covered by a Kerlix-type wrap and a second layer of Ace wrap

A 77-year-old man presented with four days of worsening swelling and malodorous drainage from chronic bilateral lower-extremity wounds. He received twice-weekly wound care but was referred to the ED because of increased exudate and concern for infection; inability to obtain timely vascular follow-up led to ED presentation. History included atrial fibrillation, diastolic heart failure, chronic obstructive pulmonary disease, hyperlipidemia, hypertension, hyperuricemia, and morbid obesity. The exam showed marked hyperkeratosis with verrucous overgrowth, bilateral lymphedema with pitting edema, erythema, and serous drainage at the ankles, with chronic venous stasis changes and malodor (Figures 1-2). Extremities were warm, nontender, and well perfused, with intact dressings, palpable pulses, capillary refill of two to three seconds, and no calf pain.

Clinical findings, timeline, and diagnostic assessment

On arrival to the ED, vital signs were stable: blood pressure 124/71 mmHg, oral temperature 36.9°C, heart rate 74 bpm, respiratory rate 18 breaths/min, and oxygen saturation 98% on room air. Initial laboratory results are summarized in Table 1.

**Table 1:** Laboratory investigations at initial presentation *Values outside the reference range. Reference ranges are those provided by the hospital laboratory reporting the results.

Test	Result	Reference range
Hemoglobin	11.2 g/dL*	13.5-17.5 g/dL
Hematocrit	34.2 %*	41-53 %
Platelet count	191 × 10³/µL	150-400 × 10³/µL
White blood cell count	8.0 × 10³/µL	4.0-11.0 × 10³/µL
Neutrophils	63.3%	40-75%
Lymphocytes	18.4%*	20-45%
Eosinophils	4.7%	0-6%
Sodium	138 mmol/L	135-145 mmol/L
Potassium	3.8 mmol/L	3.5-5.1 mmol/L
Chloride	97 mmol/L*	98-107 mmol/L
Carbon dioxide	30 mmol/L	22-29 mmol/L
Blood urea nitrogen	34 mg/dL*	7-20 mg/dL
Creatinine	1.15 mg/dL	0.7-1.3 mg/dL
Glucose	109 mg/dL*	70-99 mg/dL
Calcium	9.6 mg/dL	8.5-10.2 mg/dL
Total protein	7.7 g/dL	6.0-8.3 g/dL
Albumin	4.4 g/dL	3.5-5.0 g/dL
Aspartate aminotransferase	21 U/L	10-40 U/L
Alanine aminotransferase	11 U/L	7-56 U/
Total bilirubin	1.2 mg/dL	0.2-1.2 mg/dL
Alkaline phosphatase	99 U/L	44-147 U/L

The patient was admitted for cellulitis, with infectious disease, wound care, and surgery consulted. Outpatient management followed principles from the TIME framework: tissue optimization included bedside wound assessment, removal of dead tissue, and packing of ulcer cavities with iodoform ribbon gauze. Infection control was guided by wound cultures, which grew *Pseudomonas aeruginosa*, *Proteus mirabilis* (tetracycline-resistant), and *Staphylococcus aureus*, and he was treated with intravenous cefepime (2 g every 12 hours) for three weeks. Moisture balance was maintained with absorbent Maxorb dressings. Scheduled dressing changes continued to manage the heavy serous exudate while protecting the surrounding skin. Edge optimization focused on addressing factors impairing wound healing, including chronic venous insufficiency and lymphedema, through compression wraps, leg elevation, and coordinated outpatient wound care follow-up. Venous duplex ultrasonography demonstrated no evidence of deep vein thrombosis, and vascular surgery deferred surgical intervention at that time, recommending continued conservative wound management.

Disposition and follow‑up

The patient was discharged on day 5 with ongoing wound care, dressing changes, and follow-up with vascular surgery and wound care. Instructions included iodoform packing/Maxorb, dressing changes three times weekly, and leg elevation, avoiding adhesive due to allergy. He followed up with his primary care provider within one to two weeks and continued weekly laboratory monitoring (complete blood count and comprehensive metabolic panel).

His chronic medical conditions were continued on established regimens, including an angiotensin-converting enzyme inhibitor and furosemide for heart failure with preserved ejection fraction, ongoing management of atrial fibrillation, simvastatin for hyperlipidemia, and allopurinol for hyperuricemia. Discharge counseling included adherence to a heart-healthy diet, a goal of net negative fluid balance as clinically appropriate, weight-loss efforts, and nutrition follow-up.

## Discussion

This case describes advanced bilateral lower-extremity lymphedema complicated by ENV, severe hyperkeratosis, and chronic polymicrobial wounds. The cutaneous findings, marked hyperkeratosis, papillomatous/verrucous lesions, and woody fibrosis, are classic for ENV, an end-stage manifestation of chronic nonfilarial lymphedema from causes such as malignancy, surgery, venous disease, and obesity [3]. Chronic lymphedema predisposes to polymicrobial wounds, commonly involving *Staphylococcus aureus*, *Pseudomonas aeruginosa*, and *Proteus* species, which form biofilms that impair healing [8].

Although obesity is a commonly reported risk factor for ENV, this case is significant for the development of advanced ENV in the absence of obesity. The patient’s disease progression likely reflects the cumulative effects of longstanding lymphedema, chronic venous insufficiency, recurrent polymicrobial infection, and impaired skin hygiene. This case highlights that ENV should be considered in patients with chronic lymphatic dysfunction beyond the traditional obese population and emphasizes the importance of multimodal management, including infection control, meticulous wound care, skin hygiene, edema management, and multidisciplinary follow-up.

With concern for Gram-negative infection (including *Pseudomonas*), cefepime was started empirically and adjusted based on cultures [9]. Malodor typically arises from metabolites of anaerobic and Gram-negative bacteria. Management focuses on reducing bacterial burden and adsorbing odor using topical antimicrobials (e.g., metronidazole, silver, and iodine) and charcoal dressings, per Wound Healing Society guidelines [10,11]. Using the TIME framework (tissue debridement, infection control, moisture balance, and edge optimization) provides a structured approach to wound care by optimizing the wound bed and promoting epithelialization [12].

Other specialties that should be involved include wound care specialists for dressing changes and debridement and vascular surgery to assess perfusion, rule out ischemia, and determine if surgical intervention is needed or conservative management can continue. Perfusion is evaluated via ankle-brachial index (≥0.9) [13] and transcutaneous oxygen pressure (≥30-40 mmHg) [14]. If intervention is required for critical limb ischemia, duplex arterial ultrasound defines vascular anatomy, with normal flow triphasic or biphasic [13].

In select patients with refractory lymphedema, surgical options such as lymphaticovenous anastomosis, vascularized lymph node transfer, and liposuction may be considered. Integrating patient education into routine outpatient care has been shown to improve wound healing, reduce pain, and enhance quality of life and function. Effective management of chronic lower-extremity wounds in conditions like ENV requires a coordinated, multidisciplinary approach combining microbiology-guided therapy, wound care, edema control, education, and long-term follow-up to optimize healing, prevent recurrence, and improve outcomes.

## Conclusions

We presented a case of a 77-year-old man with longstanding lower-extremity lymphedema with severe cutaneous and infectious complications, complicated by multiple systemic comorbidities. ENV is an advanced manifestation of chronic lymphedema that often goes unrecognized. It is characterized by hyperkeratosis, fibrosis, and increased risk of polymicrobial infection. Early diagnosis and treatment are essential to minimize inflammation, infection, and impaired wound healing.

Management of this patient required a comprehensive, multidisciplinary approach, which included wound care, edema control, antimicrobial therapy, and close outpatient follow-up by primary care, infectious disease, wound care, and vascular specialists. This case highlights the importance of optimizing systemic conditions, including heart failure, obesity, and venous stasis, as well as targeted antimicrobial therapy to support wound healing. Emphasis on using the TIME framework, providing patient education, and maintaining continuous patient follow-up can significantly improve outcomes and quality of life and reduce hospitalizations in severe ENV. This case contributes to the limited research on ENV complicated by multiple polymicrobial infections and underscores the importance of early management and complex wound-care strategies.

## References

[1] Grada AA, Phillips TJ (2017). Lymphedema: pathophysiology and clinical manifestations. J Am Acad Dermatol.

[2] Manrique OJ, Bustos SS, Ciudad P (2022). Overview of lymphedema for physicians and other clinicians: a review of fundamental concepts. Mayo Clin Proc.

[3] Sisto K, Khachemoune A (2008). Elephantiasis nostras verrucosa: a review. Am J Clin Dermatol.

[4] Duckworth AL, Husain J, Deheer P (2008). Elephantiasis nostras verrucosa or "mossy foot lesions" in lymphedema praecox: report of a case. J Am Podiatr Med Assoc.

[5] Vignes S, Poizeau F, Dupuy A (2022). Cellulitis risk factors for patients with primary or secondary lymphedema. J Vasc Surg Venous Lymphat Disord.

[6] Bowler PG, Duerden BI, Armstrong DG (2001). Wound microbiology and associated approaches to wound management. Clin Microbiol Rev.

[7] Burian E, Rungby J, Karlsmark T, Nørregaard S, Cestari M, Franks P, Moffatt C (2023). The impact of obesity on chronic oedema/lymphoedema of the leg - an international multicenter cross-sectional study (LIMPRINT) (Preprint). Int J Obes.

[8] Greene AK, Grant FD, Slavin SA, Maclellan RA (2015). Obesity-induced lymphedema: clinical and lymphoscintigraphic features. Plast Reconstr Surg.

[9] Tamma PD, Aitken SL, Bonomo RA, Mathers AJ, van Duin D, Clancy CJ (2022). Infectious Diseases Society of America 2022 guidance on the treatment of extended-spectrum β-lactamase producing Enterobacterales (ESBL-E), carbapenem-resistant Enterobacterales (CRE), and Pseudomonas aeruginosa with difficult-to-treat resistance (DTR-P. aeruginosa). Clin Infect Dis.

[10] Akhmetova A, Saliev T, Allan IU, Illsley MJ, Nurgozhin T, Mikhalovsky S (2016). A comprehensive review of topical odor-controlling treatment options for chronic wounds. J Wound Ostomy Continence Nurs.

[11] Gould LJ, Alderden J, Aslam R (2024). WHS guidelines for the treatment of pressure ulcers-2023 update. Wound Repair Regen.

[12] Anesäter E, Roupé KM, Robertsson P (2011). The influence on wound contraction and fluid evacuation of a rigid disc inserted to protect exposed organs during negative pressure wound therapy. Int Wound J.

[13] Aboyans V, Criqui MH, Abraham P (2012). Measurement and interpretation of the ankle-brachial index: a scientific statement from the American Heart Association. Circulation.

[14] Yang C, Weng H, Chen L (2013). Transcutaneous oxygen pressure measurement in diabetic foot ulcers: mean values and cut-point for wound healing. J Wound Ostomy Continence Nurs.

